# Parkinson disease – Prevalence, trends and regional patterns in Germany. An analysis based on routine data from the statutory health insurance

**DOI:** 10.25646/13070

**Published:** 2025-03-31

**Authors:** Alexander Rommel, Günter Deuschl, Richard Dodel, Dinara Yessimova, Hannelore Neuhauser, Gabriela Brückner, Helmut Schröder, Katrin Schüssel, Michael Porst

**Affiliations:** 1 Robert Koch Institute, Department of Epidemiology and Health Monitoring, Berlin, Germany; 2 University Hospital Schleswig-Holstein, Kiel University, Department of Neurology, Kiel, Germany; 3 University of Duisburg-Essen, Geriatric Center Haus Berge, Essen, Germany; 4 AOK Federal Association, WIdO – AOK Research Institute, Berlin, Germany

**Keywords:** Parkinson disease, Prevalence, Time trends, Morbidity, Risk factors, Age distribution, Health claims data, Secondary data analysis, Public health

## Abstract

**Background:**

As part of the German Burden of Disease Study, population-based prevalences of important diseases are estimated. This allows regional patterns and temporal trends to be identified.

**Methods:**

The prevalence of Parkinson disease in the population was estimated cross-sectionally for the years 2017 to 2022 at the level of the Spatial Planning Regions using routine data of persons insured in the statutory health insurance AOK, adjusted for age, sex and morbidity (administrative prevalence).

**Results:**

In 2022, the prevalence of Parkinson disease in Germany was 0.35 % of the population. This represents approximately 295,000 people. The prevalence is 0.34 % in women and 0.36 % in men. The prevalence of Parkinson disease increases with age. It is 0.61 % from the age of 40 and 1.42 % from the age of 65. There is a slight downward trend over time. The age-standardised regional distribution shows no clear pattern.

**Conclusions:**

Measured by administrative prevalence, the significance of Parkinson disease for population health remains largely stable with a slight downward trend.

This article is part of a series of articles with standardised analyses for the German Burden of Disease Study of the Robert Koch Institute.

## 1. Introduction

In order to support health policy decisions, the evaluation of the burden of disease in the population is of growing importance. Burden of disease indicators represent the ‘loss’ of life years at the level of population health caused by health impairments and premature death. The methods were originally developed by the Global Burden of Disease Study (GBD) [1–3]. Disease burden indicators make it possible to compare the impact of different diseases and to draw conclusions about regional differences and trends in population health over time. As part of the German Burden of Disease Study, this methodology is adapted and applied to diseases and injuries of high public health relevance [4, 5].

In order to calculate the morbidity-related burden of disease, prevalences of diseases and injuries are needed. These alone are of great value for public health research and fill existing information gaps for diseases for which comprehensive epidemiologic descriptions are rare or lacking.

**Key messages**

► In 2022, 0.61 % of the population aged 40 and older in Germany had a diagnosed Parkinson disease.
► This prevalence was 0.57 % for women and 0.66 % for men.
► The prevalence of Parkinson disease increases significantly with age.
► The regional distribution of Parkinson disease shows no clear pattern.
► Over time, the prevalence of Parkinson disease decreased slightly from 2017 to 2022.

Although Parkinson disease has a comparatively low prevalence compared to other major noncommunicable diseases (e.g., cardiovascular diseases), it is an important cause of disease burden in the population and is particularly relevant for health and care in old age (Infobox). Only a few prevalence estimates are available for Germany, ranging from about 0.5 % in the total population to more than 2 % in people aged 65 years and older [2, 6, 7]. This article reports on the prevalence of Parkinson disease as determined by the Robert Koch Institute’s Burden of Disease Study. It is based on the standard for reporting secondary data analyses in Germany [8].

## 2. Methods

The present analysis is based on routine data of persons insured in the statutory health insurance (SHI) system. These data are mainly generated by cost accounting between service providers (e.g. hospitals) and payers (health insurance funds) in the health care system and are only subsequently made available for research purposes (secondary data analysis). Routine SHI data are collected continuously and allow trend analyses as well as small-area analyses. The data contain the most important information for estimating the prevalence: (i) diagnoses according to the 10th revision of the International Statistical Classification of Diseases and Related Health Problems, German Modification (ICD-10-GM), (ii) services according to the official classification for the coding of surgeries, procedures and general medical measures (OPS) and (iii) drug prescriptions that can be categorised using the pharmaceutical central number (PZN) of the classification according to the Anatomical Therapeutic Chemical (ATC) system [15].

The underlying methodology for calculating prevalences based on routine SHI data consists of three steps: first, the definition of the prevalence concept in the insured population (see 2.1), second, the development of the case definition for identifying diseased persons (case selection criteria, see 2.2), and third, an age-, sex- and morbidity-adjusted extrapolation of the prevalences to the whole population using regression analysis. This allows statements to be made for all residents in the regions of Germany (see 2.3).

**Infobox Parkinson disease**

Parkinson disease is the second most common neurodegenerative disease after Alzheimer’s disease. During the course of the disease, nerve cells in the brain that produce the neurotransmitter dopamine degenerate. This allows the primary Parkinson disease to be distinguished from secondary Parkinson syndromes resulting from other diseases. As a result, various disorders occur, particularly affecting motor skills [9, 10].

Typical symptoms include lack of movement (akinesia), muscle rigidity (rigor), resting tremor, and postural instability [11]. The lack of movement occurs in all people with Parkinson disease, while muscle rigidity, resting tremor, and postural instability occur in most people with Parkinson disease [9, 10]. Other possible symptoms and consequences of the disease include autonomic nervous system disorders (bladder and digestive problems, circulatory problems), memory and concentration problems, depression, and, as a result of postural instability, falls and fractures. Parkinson disease is progressive with dementia often occurring in late stages. The causes of Parkinson disease are not fully understood. In addition to age and gender (men are slightly more likely to develop the disease than women), genetic, environmental (pesticides), and medical risk factors (e.g., frequent head injuries in sports) are known [9, 10]. There are effective therapies, mainly medications, that do not cure the disease but do relieve the symptoms. The incidence of Parkinson disease does not increase significantly until after the age of 65.

Parkinson disease is a major cause of the disease burden [12]. The GBD study lists Parkinson disease among the top 20 causes of death in Germany and among the top 30 causes of years of life lost due to death [2]. In the late stages of Parkinson disease, people need assistance with many activities of daily living [9]. For this reason, Parkinson disease is one of the most common diagnoses justifying long-term care benefits under the German Social Code, Book XI [13, 14].

### 2.1 Insured population and prevalence concept for measuring 1-year prevalence of Parkinson disease

Pseudonymised routine data from around 27 million AOK insurance policyholders from the years 2017 to 2022 will be analysed using a cross-sectional approach to identify people affected by a disease [16, 17]. Prevalence is defined as the proportion of persons affected by a disease during the analysis period out of the total number of people included in the study. In analyses using routine SHI data, it should be considered that the underlying population of insured persons is an open, dynamic cohort with inflows and outflows due to natural population movements (births, deaths) or changes in an individual’s insurance history (e.g. change of health insurance company). Therefore, all calculations are not based on individuals but on observed insurance periods in days [18]. In this way, insurance periods of new-borns or deceased persons, as well as those of persons who change insurance, can be considered on a pro rata basis. The period of insurance and the regional allocation of the insured is determined on a quarterly basis. Finally, the population of insured persons, and thus the denominator of the prevalence estimate, is obtained as the total number of observed quarterly insurance periods for the respective reference year [18].

### 2.2 Case definition for Parkinson disease

A case definition for the inclusion of persons with prevalent Parkinson disease has been developed in collaboration with recognised internal and external experts. The period analysed always refers to twelve months. Selection criteria are based on ICD-10-GM coded diagnoses and prescribed drugs (ATC codes) from the following areas of health care delivery (Table 1).

Since Parkinson disease can occur in children and adolescents, albeit very rarely, all persons in the insured population were considered without age restrictions. For the purpose of considering primary Parkinson disease, the ICD-10 codes G21 – G23 were used to define exclusion diagnoses that would rather be assigned to ‘other neurological disorders’ in the calculation of the disease burden (Infobox). Even if a G20 code was present at the same time, such cases were interpreted as unclear diagnoses and not included in the affected group. The criteria were applied to all persons in the insured population in each quarter of the reference year, looking back three quarters from the reference quarter to determine 1-year prevalence. Finally, to determine the number of persons affected by a disease and thus the numerator of the prevalence calculation, the observed person-time of the cases in each quarter of a calendar year was summed up.

### 2.3 Statistical methods

Since the group of policyholders of a health insurance fund is not a random sample of the general population and is therefore not representative of the population [17, 19–22], the specific prevalence estimates for each health insurance fund must be extrapolated to the whole population. Due to the regionally different distribution of the population in each health insurance fund, this extrapolation is done by region [23]. In this regression analysis, regionally available statistics on the frequency of inpatient diagnoses and on the demographic structure of the population are used as auxiliary information. In this way, in addition to demographic differences, morbidity differences between health insurance funds and the German population can be corrected (morbidity-adjusted) and differentiated by small areas. The method was developed and its plausibility tested using type 2 diabetes as an example [23]. It has been adapted for Parkinson disease to estimate the prevalence for the whole population of Germany at the level of the 96 Spatial Planning Regions for Parkinson disease for each reference year.

When extrapolating prevalences, individual age groups are combined into larger age groups for model stability, so that a prevalence is not always available for each 5-year age group. To allow stratification at this level of detail, a special procedure is used to model missing age-specific prevalences. For this purpose, the sex-specific prevalence patterns of the AOK population along the 5-year age groups (raw data) are transferred to the (pooled) age groups of the extrapolation. The extrapolated total prevalence in the combined age group serves as the target value for the modelling. The statistical uncertainty is derived from the variance of the morbidity-adjusted total prevalence. In addition, the results are age-standardised using the European Standard Population 2013 [24] for the presentation of maps and time trends.

## 3. Results

**Figure 1:**
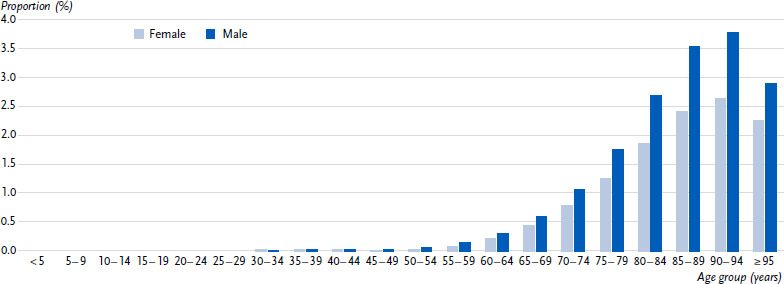
Prevalence of Parkinson disease by age and sex (% of population). Source: Burden of Disease Study for Germany (AOK routine data 2022, age-, sex- and morbidity-adjusted and extrapolated to the German population)

Based on the case definition used, 0.35 % of the population in Germany is affected by Parkinson disease in 2022 (administrative prevalence). This corresponds to nearly 0.3 million people. The prevalence of Parkinson diseases increases significantly with age. From the age of 40, the prevalence is 0.61 %, which is 0.57 % for women and 0.66 % for men. For those aged 65 and over, the prevalence is 1.42 %. It reaches its highest value in the 90 to 94 age group at 2.64 % for women and 3.79 % for men. The prevalence decreases again from the age of 95. Early onset of Parkinson disease under the age of around 40 is very rare. The gender difference in favour of a higher prevalence among men is maintained throughout the age course (Figure 1, Annex Table 1).

**Figure 2:**
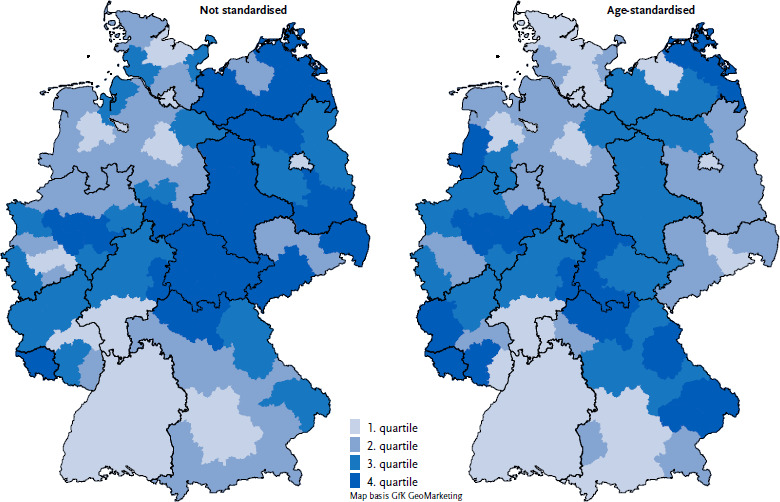
Prevalence of Parkinson disease on the level of the Spatial Planning Regions (% of population, quartiles). Source: Burden of Disease Study for Germany (AOK routine data 2022, age-, sex- and morbidity-adjusted and extrapolated to the German population)

The regional distribution of the prevalence of Parkinson disease shows no clear pattern. The impression of a higher crude prevalence in eastern Germany is mainly an effect of the older population in eastern Germany. This pattern disappears after age standardisation (Figure 2). A rather low prevalence is found in some regions of Schleswig-Holstein, Baden-Württemberg and southern Bavaria. A rather high prevalence is found in some regions of North Rhine-Westphalia, Thuringia, Saarland, Rhineland-Palatinate and in the north and east of Bavaria.

**Figure 3:**
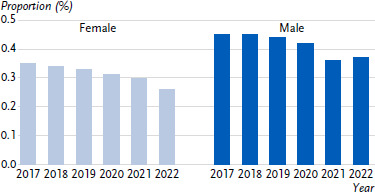
Prevalence of Parkinson disease over time (% of population, standardised by age). Source: Burden of Disease Study for Germany (AOK routine data 2017–2022, adjusted for age, sex and morbidity and extrapolated to the German population)

The age-standardised prevalence of Parkinson disease shows a decreasing trend over time. In 2017, the overall prevalence of 0.38 % was 0.09 percentage points higher than in 2022 (Figure 3, Annex Table 2).

## 4. Discussion

In Germany, 0.35 % of the population has been diagnosed with Parkinson disease. Parkinson disease is more common in men than in women and is strongly associated with age by increasing significantly from around retirement age. The prevalence is 0.61 % from the age of 40 and 1.42 % from the age of 65. At younger ages, the disease is very rare. The regional distribution does not show a clear pattern. There has been a slight decrease in the prevalence of Parkinson disease over time.

The findings on the age and sex distribution of the prevalence of Parkinson disease are broadly consistent with the findings in the research literature based on routine health care data [6, 7, 25, 26]. The declining age-standardised incidence of the disease over time is particularly noteworthy. The significant increase in age-standardised Parkinson disease prevalence of about 20 % between 1990 and 2016 observed in the GBD study [27] could lead to the expectation that Parkinson disease prevalence will continue to increase in Germany. However, various analyses based on claims data also show a decline in age-standardised prevalence [6, 7, 28] and incidence [7, 28, 29], which in some studies is more pronounced in women. Increasing, stagnating, and decreasing Parkinson disease incidence rates have been reported internationally [29]. There is still no conclusive explanation for the decline in the incidence of the disease. It is possible that the decline in certain risk factors, e.g. environmental factors such as pesticide exposure or changes in dietary habits, may explain part of this development [7, 29, 30]. As the present study is an evaluation of claims data, a change in the diagnostic and coding behaviour of treating physicians may also be a cause [7]. In principle, the COVID-19 pandemic could influence the diagnosed prevalence by reducing contact with the health care system. However, short-term reduced physician visits with subsequent catch-up effects may not necessarily affect the 1-year prevalence if it is based on robust case definitions (see below).

It is striking that age- and sex-specific prevalences based on other routine data analyses are usually somewhat higher [6, 7, 26]. In addition to the decreasing trend, this can also be explained by differences in case definitions. In the present study, only the ICD-10 code G20 and no other diagnoses were considered as inclusion criteria. In addition, a relatively broad exclusion criterion of secondary Parkinson disease with diagnoses G21, G22, or G23 was applied. A meta-analysis of European primary data surveys shows a significantly lower prevalence of 0.22 % for the year 2013 in Germany [31]. However, the authors note that prevalence estimates based on medical records may be underestimated [31]. Regarding regional patterns, another routine data analysis also found low prevalence in Baden-Württemberg and southern Bavaria and higher prevalence in some districts of Thuringia, north-eastern Bavaria, Saarland and Rhineland-Palatinate [7].

Compared with the GBD study estimates, the present prevalence is lower (0.52 % vs. 0.35 %) [27]. However, the GBD estimates for Germany are comparatively high and show a continuing upward trend. The GBD prevalence for Germany is about twice that of the USA. As the GBD study is based on modelling from a variety of international data sources, these differences are difficult to explain. The discrepancies regarding a possible overestimation of the Parkinson disease prevalence for Germany in the GBD study should be further clarified in future studies.

The present analysis relies on routine SHI data. One advantage of such data is that some of the typical sources of error associated with primary data collection, such as surveys, are excluded. These include bias due to recall bias, non-response or lower participation of hard-to-reach groups [32]. One limitation that needs to be considered is that SHI routine data mainly contain information relevant for accounting (see 2.). Non-utilisation of health services, lack of documentation of diagnoses and financial incentives to optimise accounting can lead to misclassification and bias in the data [32, 33]. Non-utilisation is of little relevance for many conditions if they are so severe, such as strokes, that they usually lead to medical contact or hospitalisation. However, misclassification (over-or underestimation) of diseased persons can occur if diagnoses are coded incorrectly or not at all.

For Parkinson disease, there may also be an underreporting of mild cases because the disease is often diagnosed some time after the onset of symptoms. In addition, diagnostic differentiation from other, much rarer diseases can be difficult, especially at the onset of the disease (especially atypical parkinsonism). In order to minimise misclassifications in routine data, disease-specific case definitions were developed for each disease, which, in addition to diagnoses, use further information on surgeries, drug prescriptions or outpatient claims codes for plausibility checks [18, 33, 34].

For example, outpatient Parkinson disease diagnoses were internally validated using the M2Q criterion (diagnosis in at least two quarters during the analysis period) or the presence of a medication prescription. The latter may have led to some cases not being finally confirmed, as medications are often prescribed when Parkinson disease is first suspected, but their clinical effect only may confirm the presence of Parkinson disease. However, sensitivity analyses conducted as part of the present project show that the relative increase in prevalence due to the inclusion criterion of a single diagnosis in conjunction with a drug prescription was in the low single-digit percentage range, and therefore there is no clear overestimation of Parkinson disease prevalence due to this criterion. In addition, all diagnoses indicating other causes of Parkinson symptoms were excluded (Secondary parkinsonism, Parkinsonism in diseases classified elsewhere, other degenerative diseases of basal ganglia).

Other limitations of the results are related to the statistical methods used for extrapolation and for modelling the age distribution in the 5-year groups. The extrapolation method uses the diagnoses of all hospital admissions in Germany to adjust for differences in morbidity between insurance funds and the population, and has been developed and validated for type 2 diabetes [23]. It is thus assumed that the estimated prevalences no longer reflect the insurance fund specific morbidity, but that of the population. To model age distribution, it was assumed that the age progression of Parkinson disease among AOK insured persons could be applied to the combined age groups from the extrapolation results. The overall prevalence remains unaffected by this procedure. To assess the plausibility of extrapolation and age modelling, the results were compared with published values for the prevalence of Parkinson disease from Germany whereby the deviations, as shown above, were small or can be attributed to methodological differences in the case definitions [6, 7, 25, 26, 31, 35].

Burden of disease studies place high demands on the data to be used. Among other things, they require the most accurate information possible on the frequency of disease by age, sex and region. For many diseases, routine data from the SHI system are the preferred option for estimating and presenting prevalence at the small area level. Thus, burden of disease studies, especially when conducted regularly, provide important basic epidemiological information and fill information gaps. Parkinson disease is a disease of high public health and health care relevance due to its frequency and treatment demand and is a major cause of disease burden in the population. Parkinson disease is associated with many comorbidities, including dementia and depression [7], and usually results in the need for long-term care [13]. At the same time, there is evidence of a lack of medical care for people requiring inpatient care, including many people with Parkinson disease [36]. This highlights the need for coordinated multidisciplinary care, including outpatient and inpatient care. Further research is needed to clarify the reasons for the decline in prevalence. Epidemiologic changes in Parkinson disease risk factors should be considered, as well as possible changes in the diagnostic and coding practices of physicians [7].

## Figures and Tables

**Table 1:** Selection criteria for defining the prevalence of Parkinson disease with AOK routine data

Health care sector	Inpatient care^1^	Outpatient care		
		Specialised ambulatory care^2^	Ambulatory care in medical practices^3^	
**Inclusion criteria**				
**Criterion**	At least one diagnosis in the analysis period	Diagnosis in at least two quarters in the analysis period^4^	OR	Diagnosis AND medication in the analysis period
**Codes**	ICD-10-GM^5^: G20 Parkinson disease			
	ATC^6^: N04 Anti-parkinson drugs			
**Exclusion criteria**				
**Criterion**	At least one diagnosis in the analysis period			
**Codes**	ICD-10-GM diagnosis: G21 Secondary parkinsonism G22 Parkinsonism in diseases classified elsewhere G23 Other degenerative diseases of basal ganglia			

^1^ Inpatient cases (§ 301 para. 1 SGB V): Main and secondary diagnoses of the complete inpatient and day patient cases (discharge diagnoses)

^2^ Cases of specialised ambulatory care (§§ 115b, 116b, 117 para. 1 to 3, 118, 119, 119c, 120, 140a SGB V) (mainly ambulatory care in hospitals)

^3^ Cases of ambulatory care in medical practices paid under the scheme of statutory health insurance (§ 295 para. 2 SGB V)

^4^ So called M2Q-criterion

^5^ Inpatient: main OR secondary diagnoses; outpatient: diagnostic code ‘secured’

^6^ Prescription in the pharmaceutical accounting data under § 300 para. 1 SGB V

ICD-10-GM = International Statistical Classification of Diseases and Related Health Problems, 10th Revision, German Modification, SGB = Social Security Code, ATC = Anatomical Therapeutic Chemical classification system

**Annex Table 1:** Prevalence of Parkinson disease by age and sex (percentage of population). Source: Burden of Disease Study for Germany (AOK routine data 2022, adjusted for age, sex and morbidity and extrapolated to the German population)

Age group (years)	Female	Male	Total
	%	%	%
0–4	0.00	0.00	0.00
5–9	0.00	0.00	0.00
10–14	0.00	0.00	0.00
15–19	0.00	0.00	0.00
20–24	0.00	0.00	0.00
25–29	0.00	0.00	0.00
30–34	0.02	0.01	0.01
35–39	0.03	0.02	0.02
40–44	0.03	0.02	0.02
45–49	0.01	0.03	0.02
50–54	0.02	0.06	0.04
55–59	0.07	0.14	0.11
60–64	0.21	0.31	0.26
65–69	0.44	0.6	0.51
70–74	0.78	1.07	0.91
75–79	1.25	1.76	1.48
80–84	1.86	**2.7**	2.21
85–89	2.41	3.54	2.84
90–94	2.64	3.79	2.99
≥95	2.26	2.92	2.41

**Annex Table 2:** Prevalence of Parkinson disease over time (% of population, crude and age-standardised). Source: Burden of Disease Study for Germany (AOK routine data 2017 – 2022, age-, sex- and morbidity-adjusted and extrapolated to the German population)

Year	Female (not standardised)	Male (not standardised)	Total (not standardised)	Female (age-standardised)	Male (age-standardised)	Total (age-standardised)
	%	%	%	%	%	%
2017	0.38	0.39	0.39	0.35	0.45	0.38
2018	0.38	0.39	0.39	0.34	0.45	0.37
2019	0.37	0.40	0.36	0.33	0.44	0.36
2020	0.36	0.39	0.37	0.31	0.42	0.35
2021	0.35	0.37	0.36	0.30	0.36	0.30
2022	0.34	0.36	0.35	0.26	0.37	0.29
